# Predicting the critical organizational behavior and culture of the Turkish construction industry's occupational groups for determining the success of the construction business

**DOI:** 10.1016/j.heliyon.2024.e33197

**Published:** 2024-06-18

**Authors:** Muhammed Ernur Akıner, İlknur Akıner, İbrahim Yitmen

**Affiliations:** a Vocational School of Technical Sciences, Department of Environmental Protection Technologies, Akdeniz University, 07058, Antalya, Turkey; b Department of Architecture, Faculty of Architecture, Akdeniz University, 07058, Antalya, Turkey; c Department of Construction Engineering and Lighting Science, Jönköping University, 551 11, Jönköping, Sweden

**Keywords:** Contracting companies, Culture in construction, Factor analysis, Organizational behavior, Questionnaire

## Abstract

Organizational culture is connected to people's capacity to organize themselves internally and adapt to the outside environment. Contracting companies were asked to complete a questionnaire based on organizational culture characteristics. This study intends to make a substantial theoretical contribution by demonstrating a broader perspective on exposing the essential cultural features using a quantitative approach with the fewest possible questions. Exploratory factor analysis and Principal Component Analysis were used to identify the most critical variables and extract a more manageable number of components from multiple related variables. Twenty-eight variables were reduced to thirteen to eliminate statistical cross-loading. Furthermore, the organizational culture was simplified from eight to three dimensions. Thirteen factors were created based on cultural habits in the construction sector, with three inclusive dimensions: multiple motivational, one–bipolar, and many bipolar. This study provides a more succinct survey that quantifies cultural habits and their influence on sectoral performance.

## Highlights

• The study discovered a substantial statistical link within the questionnaire items used to evaluate corporate culture's impact on performance.
• The study disclosed that the critical cultural aspects can be surveyed using a quantitative technique with the fewest possible questions.
• Exploratory factor analysis and Principal Component Analysis were applied to extract a more manageable number of factors.
• Twenty-eight variables were reduced to thirteen, and the organizational culture was reduced from eight to three dimensions.
• Upon the cultural behaviors, thirteen variables were defined under dimensions: multiple motivational, one bipolar, and many bipolar.

## 1 Introduction

Culture has been a prominent discussion, debate, and inquiry issue in the construction industry during the last decade. It is a collection of common experiences, interpretations, and attitudes shared by members of a group, organization, community, or country [1,2]. Culture is a deeply embedded behavioral characteristic that affects how firms approach, assess, and negotiate international business possibilities [3,4]. Various cultures lead companies to have different management paradigms and conceptions like national, organizational, and project cultures.

Regardless of the diversity of the employees, the company culture governs their collective conduct. Culture is a pattern of fundamental assumptions dealing with external adaption and internal integration difficulties. Cultural phenomena in organizational cultures include artifacts and behaviors, visible organizational processes, espoused values, goals and philosophies, assumptions, feelings, and attitudes [[5], [6], [7]]. The construction industry's organizational culture reflects project-based, subsequent, and temporary arrangements.

Culture primarily concerns the differences in people's conduct in all aspects of the construction industry's operations. Bourdieu [8] established a framework for accessing culture and examining its repercussions. In literature, the symbolic spheres (which may be explored using semiotics) are roughly depicted as strata of human behavior, or "habitus." This term describes how individuals behave and interact with one another. People's activities are essential, and Bourdieu [8] contributes to the motivations behind various behaviors.

Employee attitudes, organizational effectiveness, market performance, and financial success are influenced by organizational culture [9,10]. Organizational strategy and structure affect knowledge management and organizational efficiency [11,12].

This study focused on the organizational behavior and culture of critical stakeholders in Turkish contracting companies. This study also highlights and enhances cultural diversity across contracting firms to identify construction project participants' cultural characteristics and orientations. Finally, the technique in this research aims to offer suggestions to construction decision-makers or engineering managers who want to systematically adopt most of the results to other project-based contractor organizations.

There are many issues and difficulties facing the culture of the construction business that need to be addressed. One of the main problems is the industry's lack of innovation and reluctance to change. Many companies continue to function with antiquated ideas and archaic methods, which impede efficiency and advancement [13]. Other issues include a lack of diversity and the construction sector's pervasive "macho" mentality [14]. This makes the workplace unwelcoming for women and members of minority groups, in addition to restricting the industry's capacity to draw in new talent. The industry's potential for expansion and the retention of personnel is restricted by the persistence of discrimination and harassment [15].

In this research, the non-innovative and traditionalist "macho" culture issue in the construction business is connected to Questions (Q) Q7 and Q9, per the factor analysis findings and principal component analysis in Table 6. This inhibits creativity and innovation and maintains a limited perspective that might make it more difficult for the sector to adjust to shifting market dynamics [16]. According to Anjum et al. [17], this "toxic culture" influences staff morale and productivity and can lead to high turnover rates. Q14, Q20, Q22, and Q27 are the questions to identify the problem of toxic cultures in the construction industry.

The continued use of a "top-down, hierarchical management style" is another issue, as it obstructs efficient cooperation and communication. The industry's image is weakened due to the frequent delays, cost overruns, and lack of transparency [18]. Q1, Q16, Q24, and Q25 are the questions related to the construction business management issue.

Furthermore, the building industry's culture values short-term benefits over "long-term sustainability." This mentality undermines environmental stewardship and the implementation of sustainable practices. The industry must adopt sustainable construction processes and materials to reduce environmental impact [19]. Another issue is the resistance to change or adapt to new technologies and practices. Many construction companies still rely on traditional methods and resist incorporating new advancements, such as Building Information Modeling (BIM) or sustainable construction practices [20]. Regardingly, the construction sector has been slower in embracing digitalization and automation compared to other industries, which hampers efficiency, increases costs, and hinders competitiveness [21]. Lastly, the construction industry's culture has slowly adopted technological advancements and digitalization [22]. This reluctance to embrace technology hinders productivity and limits the potential for innovation and growth [23].

For the construction industry to succeed in the long run, overcoming reluctance to change and cultivating an innovative culture will be imperative. All parties involved in the building sector must work together to address these issues. The building sector has to aggressively foster a culture of "innovation," "inclusivity," "sustainability," and "adaptability" to solve these issues [24]. Long-term success in the sector will depend on fostering a culture of constant learning and development, adopting sustainable practices, and supporting diverse talent and viewpoints. It will be necessary to take proactive measures to achieve this, such as funding training initiatives, enacting diversity and inclusion laws, encouraging environmentally friendly behavior, and embracing technology developments. The construction sector can only really progress and prosper in the future by tackling these cultural obstacles. Transforming the industry's culture and guaranteeing its sustainability in the face of changing challenges requires focusing on diversity and inclusion, promoting an inclusive and respectful work environment, and investing in technology innovations. Through this approach, the sector may tackle cultural obstacles and create opportunities for a more sustainable and profitable future. The long-term sustainability, innovation, integration, and adaptability challenges facing the construction industry are covered in questions Q6, Q17, and Q21.

## 2 Theoretical background

### 2.1 The construction Industry's organizational behavior and culture

Artifacts [5], modes of behaving [25], and work practices [26] are all components of organizational culture. Understanding the link between organizational performance and culture is vital to achieving desired organizational results [27,28]. It's widely accepted that an organization's culture significantly impacts its performance [29,30]. Since the construction sector relies so significantly on human power, it is regarded as having an excellent chance to improve its current level of performance via its organizational culture [30,31]. Kimbrough and Componation [32] pointed out that organizational culture incorporates significant initiatives where the firms can respond to market changes expediently and whether the construction firms can successfully overcome substantial developments.

According to experts, establishing a solid corporate culture is critical to a company's success. According to Yazici [33], organizational culture significantly impacts the performance of individuals or teams.

People from various backgrounds manage construction projects, resulting in a wide range of human behavior and project expectations [34]. The European Union's (E.U.) construction industry is primarily attributed to an adequately supplied and robust organizational culture that recognizes and celebrates the cultural backgrounds of its workforce. This can be achieved, for instance, by upholding culturally significant events within the company and allowing all employees, regardless of ethnicity, to retain their unique cultural identities [35].

According to Serpell and Rodriguez [36], vital cultural aspects of construction enterprises may be influenced by strategic actions. Project participants must understand how culture develops and impacts to maximize its benefits. Teräväinen et al. [37] found a correlation between Finnish construction projects' efficiency and organizational culture. The construction projects' current and ideal culture profiles were analyzed in depth, including the links between project culture, customer satisfaction, and project success. The planned cultural change in building projects seemed to diverge significantly from the desired transformation in the Finnish construction sector. Teräväinen et al. [38] claim that an Adhocracy-like culture is more prevalent in the construction industry than a hierarchical culture. Diverse leadership styles are required at various organizational levels and working contexts, even within the same industry or business. However, a detailed examination of the project's features' readiness to adopt the intended cultural change is required.

This paper provides a correct approach to defining culture, arguing that the Turkish construction sector will only advance and flourish if cultural barriers are overcome.

### 2.2 Evaluation of the organizational behavior of the construction industry

Understanding the forms of conduct on which culture has the most significant influence and how culture influences contracting business members' behavior. For example, Alfons Haar, a German construction company, argues that its top priorities are maintaining a positive image and providing outstanding service. Therefore, there is a need to understand culturally appropriate behavior in different cultures.

In the same way that an evaluation of forces evaluates elements like size and direction, an investigation of cultural constructions identifies qualities essential to the culture. These characteristics are known as "organizational culture dimensions," and "hardening" the notion of organizational culture entails identifying these dimensions and generating empirical referents that can be measured around them.

Power distance, uncertainty avoidance, masculinity/femininity, and individualism/collectivism were used by Hofstede [25] to build a "value survey module." Hampden-Turner and Trompenaars [39] mainly concentrated on the dimensions of achieved/ascribed status, individualism/communitarianism, inner-directed/outer-directed, long-term orientation, masculinity/femininity, power distance, uncertainty avoidance, and universalism/particularism. Schein [40] also noted time, space, human nature, human activity, and relationships. According to Quinn [41], the "competing values framework" highlighted the importance of leadership skills, organizational environment, success criteria, and management style.

Quinn [41] compiles a list of cultural elements developed by researchers interested in the subject. Hofstede [42] considered the IBM survey's most critical national culture characteristics, in which cultural dimensions were specified experimentally based on power distance and uncertainty avoidance. Hampden-Turner and Trompenaars [39] and Hampden-Turner and Trompenaars [43] proposed two dimensions analogous to Hofstede's: equivalence/hierarchy and person/orientation.

When these varied contributions are added, many dimensions may be compiled to represent the study views. According to Ankrah and Langford [44] and Hofstede [25], having too many dimensions in a measuring framework loses relevance by being difficult to understand. As a result, this research required defining a few key aspects that would serve as the foundation for the ensuing evaluation. As Hofstede proved, this dimension identification may haphazardly depend on the research's goals [25].

To analyze a culture's identity, aspects that define the structure must be identified. The quantity and direction of these factors are the most important [25]. Aspects of culture are the terms used to describe these challenges. The concept of organizational culture necessitates the identification of numerous dimensions, which can then be measured based on a range of circumstances, resulting in empirical reference points. Attention is often paid to human behavior, manufacturing processes, human relationships, managerial characteristics, technology, organizational learning, innovation, and environmental issues.

According to Hofstede [42], the best technique for culture research is a conciliation strategy incorporating qualitative and quantitative approaches. This study used quantitative methods for Turkey, where there is a scarcity of culture studies on organizational culture in construction in contracting firms. As a result, the quantitative portion of the study included this article to get an early influence on the cultural aspects of contracting firms. On the other hand, the qualitative nature of this research is its limitation. As pilot research with some organizations, the qualitative phase was undertaken only to modify and select the variables.

Organizational culture may impact employees' identification with their country's culture. Organizational culture highly influences participants' values, and national origin reveals itself in organizational behavior [25]. According to Schein [5], the principal contractor, client, investor, subcontractors, suppliers, civil engineer, and architect are the primary makers of organizations and organizational culture. The aggregation of individual opinions is the result of the company culture. Particularly in Turkey, the contractor is the primary player in the culture's growth.

## 3 Methodology

### 3.1 Sampling

**Fig. 1:**
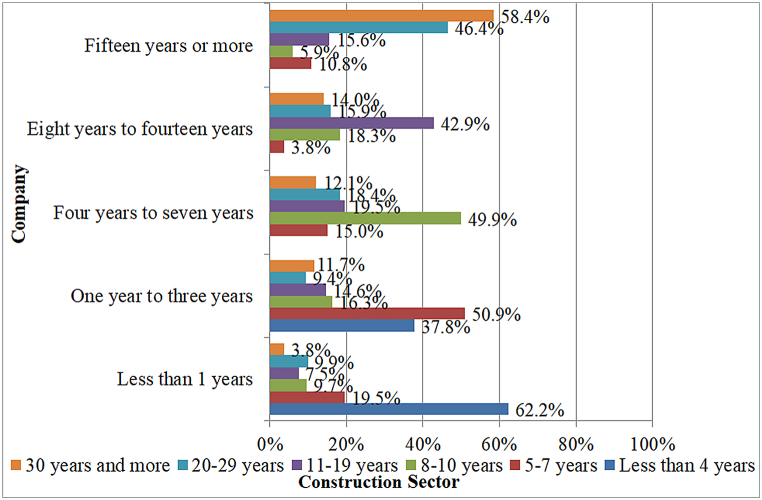
How long has a survey respondent worked for their organization, broken down by how long they have been in the construction business.?.

This research focuses on the Turkish Contractors Association (TCA), a registered contracting enterprise. The poll focused on Istanbul, Ankara, Izmir, Bursa, and Antalya since these cities have the most people and are the most actively developing in building. The chamber of commerce and TCA provided a list of contracting enterprises active in the construction industry, totaling 600 companies—the companies in the sample range in size from small to medium. Small and medium-sized businesses comprise the bulk of the Turkish construction sector [45]. Table 1 shows the profile of the respondents in the contracting firms in percentage. Fig. 1 depicts how long a survey participant has been employed by their organization, divided into categories based on how long they have been in the construction sector.

**Table 1:** The profile of the respondents in the contracting firms in percentage.

		Frequency	Percent	Cumulative Percent
**City**	Ankara	55	11.0	11.0
	Bursa	10	2.0	13.0
	İstanbul	348	69.6	82.6
	İzmir	82	16.4	99.0
	Antalya	5	1.0	100.0
	Total	500	100.0	
**Gender**	Male	440	88.0	88.0
	Female	60	12.0	100.0
	Total	500	100.0	
**Age**	Age between 20 and 29	120.0	24.0	24.0
	Age between 30 and 39	142.0	28.4	52.4
	Age between 40 and 49	130.0	26.0	78.4
	Age between 50 and 59	76.0	15.2	93.6
	Age 60 or over	32.0	6.4	100.0
	Total	500	100.0	
**Title**	Owner of the company	246	47.0	47.0
	Civil Engineer	46	11.4	58.4
	Building Architect	32	6.4	64.8
	Contractor	23	4.6	69.4
	Manager, Director	96	19.2	88.6
	Technician	16	3.2	91.8
	Site Manager	6	1.2	93.0
	Electrical Engineer	4	0.8	93.8
	Survey Engineering	3	0.6	94.4
	Mechanical Engineer	7	1.4	95.8
	Landscape Architect, Urban Planner	7	1.4	97.2
	Interior Architect	2	0.4	97.6
	Engineer	1	0.2	97.8
	Business Development	4	0.8	98.6
	Other	9	1.4	100.0
**Number**	1 Employee	35	7.0	7.0
	2 to 10 Employees	280	56.0	63.0
	11 to 25 Employees	108	21.6	84.6
	26 to 99 Employees	64	12.8	97.4
	100 and over	13	2.6	100.0

### 3.2 Data collection

The data was gathered via a questionnaire sent to all registered TCA businesses. Participants were informed of the thoroughly scientific and private study's objectives, and anonymity was guaranteed. The study included 600 respondents from distinct firms (one respondent per company), and 500 questionnaires were received, yielding an 83 percent (500/600) response rate. In December 2021, the quantitative approach (face-to-face survey) was finished. Before the survey's conclusion, interviewees were chosen at random.

The variables, in other words, questionnaire questions, aim to reveal the impacts of regulations, hierarchy, gender equality, business planning, collaboration, market adaption, risk-taking, and qualifications. Depending on their organizational structures, various businesses display specific behaviors. In addition, the participants' ideas and behavior differed based on the variant cultural characteristics, and the perspective also changed depending on the respondent's position. For example, a company's managers, top managers, and experts may respond in various ways.

The dimensions and variables employed in this study were established in two phases. First, eight dimensions of the organizational culture and the 28 independent variables were grouped based on achieved/ascribed status, individualism/communitarianism, inner-directed/outer-directed, long-term orientation, masculinity/femininity, power distance, uncertainty avoidance, and universalism/particularism. Table 2 lists the 28 questions that were included in the questionnaire.

**Table 2:** A questionnaire to gain a better understanding of contracting firms' organizational behavior.

Dimension 1. Universalism/Particularism [39,43]					
*The regulations are discussed in this section. Please consider any rules that may exist in your organization. Universalism emphasizes the importance of adhering to norms, whereas particularism emphasizes the importance of exceptions and the possibility of deviant behavior.*	Most	Very	Moderately	Little	Very little
Question 1 (Q1). There are written, unwritten, and informal regulations in our organization. Every co-worker is aware of them and expects others to follow their lead.	1	2	3	4	5
Q2. While specific guidelines may exist, personal interactions between co-workers enable more effective issue solutions.	1	2	3	4	5
Q3. Some colleagues at our firm are less respectful of rules than others, but no one complains as long as they perform a decent job.	1	2	3	4	5
**Dimension 2**. **Power Distance** [25,46]					
*Hierarchy is the focus of this section. First, please consider your company's organizational structure. A high power distance indicates significant levels of command, while a low power distance indicates that power and influence over choices are more evenly distributed across colleagues in your firm.*	Most	Very	Moderately	Little	Very little
Q4. Our organization has a defined structure with various distinct management levels.	1	2	3	4	5
Q5. Superiors make decisions in our firm, and they seldom inquire about the opinions of their subordinates.	1	2	3	4	5
Q6. It is uncommon to change the company's future or how a particular function is performed, leaving co-workers astonished.	1	2	3	4	5
**Dimension 3**. **Masculinity/Femininity** [25,46]					
*Gender equality is the theme of this section. Please consider your female co-workers, the positions they have in your firm, the rights women have to participate in conversations concerning the company's future or job completion, and, as far as you know, their compensation.*	Most	Very	Moderately	Little	Very little
Q7. Equally competent women do the same tasks as men.	1	2	3	4	5
Q8. Female co-workers earn at least as much as their male counterparts in our firm.	1	2	3	4	5
Q9. Working alongside a male or female colleague, whether superiors or subordinates, makes no difference.	1	2	3	4	5
Q10. We have a competitive rather than cooperative relationship with co-workers.	1	2	3	4	5
Q11. To take additional risks to get a higher-paying job.	1	2	3	4	5
Q12. To have difficult things to do that give you a feeling of personal success.	1	2	3	4	5
**Dimension 4**. **Uncertainty Avoidance** [25,46]					
*Taking risks is the topic of this section. But, first, please consider your company's obligations and liabilities. Because of their unique attitude of avoiding uncertainty, colleagues at all levels of management, for example, seldom make choices on their own. The polar opposite is a mentality in which the ability to make rapid decisions is prized more highly and makes errors more readily accepted.*	Most	Very	Moderately	Little	Very little
Q13. Individuals are given clear guidelines as to how they should plan their daily tasks. In addition, the roles and responsibilities are clearly defined.	1	2	3	4	5
Q14. Our workers are concerned about their supervisors' reactions to their everyday jobs, particularly in unusual circumstances.	1	2	3	4	5
Q15. In our firm, employees trust one another and do not need to supervise them constantly.	1	2	3	4	5
Q16. It is often vital to develop documented formal processes to ensure the completion of the project.	1	2	3	4	5
Dimension 5. Long Term Orientation [25,46]					
*This section discusses your company's planned horizon. Please consider your company's investment, expenditure, and staffing policies. A long-term mindset implies that the economy and reserves are vital compared to high profits or earnings and minimal staff turnover.*	Most	Very	Moderately	Little	Very little
Q17. Only essential and long-lasting equipment is purchased in our organization. Therefore, employees are asked to use caution while using them.	1	2	3	4	5
Q18. Employees are hesitant to quit our organization, even if they can earn a little more elsewhere.	1	2	3	4	5
Q19. Debts are avoided in our firm.	1	2	3	4	5
**Dimension 6**. **Individualism/Communitarianism** [39,43]					
*This section is about teamwork. First, consider how your co-workers interact with one another in your workplace. Individualism suggests individuals have different personality traits, while communitarianism says that the group's overall performance is more essential than individual employees' attitudes and behaviors.*	Most	Very	Moderately	Little	Very little
Q20. We get the most remarkable outcomes at our organization by working together.	1	2	3	4	5
Q21. Some of our staff are unique individuals with unusual attitudes and working styles.	1	2	3	4	5
Q22. Employees in our organization discuss difficulties and keep their co-workers up-to-date on significant developments.	1	2	3	4	5
**Dimension 7**. **Inner-Directed/Outer-Directed** [39,43]					
*The topic of market adaptability is the focus of this section. Please consider how your organization responds to market changes. Inner-directed indicates that your firm creates ideas and plans with its employees, but outer-directed suggests that your organization is readily impacted by information from outside sources.*	Most	Very	Moderately	Little	Very little
Q23. Our firm does not need to keep up with every market trend.	1	2	3	4	5
Q24. Tax advisors or management consultants significantly impact our company's growth.	1	2	3	4	5
Q25. Colleague's suggestions for improvement are greatly appreciated.	1	2	3	4	5
**Dimension 8**. **Achieved Status/Ascribed Status** [39,43]					
*The purpose of this section is to discuss qualifications. Please consider the education of your company's employees. Employees in your organization are promoted based on the quality of their work, referred to as "achieved status." Dealings, relationships, a good reputation, or certain rights play a more prominent role in Ascribed Status.*	Most	Very	Moderately	Little	Very little
Q26. Our organization's positions are exclusively given to those with the highest credentials and talents.	1	2	3	4	5
Q27. Some of my co-workers have risen through the ranks due to their experience.	1	2	3	4	5
Q28. Individuals get employed based on their excellent connection with a supervisor, not their performance ability.	1	2	3	4	5

Independent variables were also obtained and recreated for the second stage from many publications, including the questionnaire implemented by Hofstede et al. [46], Hofstede [25] and dimensions referred to by Hofstede et al. [46], Hofstede [25], Hampden-Turner and Trompenaars [39,43] and Freeman and Bordia [47]. However, to customize the survey to the specificities of contracting organizations, most of these characteristics were constructed based on the outcomes of the pilot business interviews. Minor changes to variables had little effect on the design, and constructs were already considered validated. The five-point Likert scale rated the respondents' level of agreement or disagreement on a scale ranging from 1 to 5 as "Most," "Very," "Moderately," "Little," and "Very little."

### 3.3 Factor analysis

Factor analysis is a correlational strategy used to uncover and explain the underlying variables driving data values for a broad group of values [48]. Factor analysis finds relationships between and among variables to group them into a single underlying factor that drives their values. For this reason, factor analysis is often referred to as a data reduction process to determine if a factor is essential, and then the interaction of the variables with the factors is examined [49]. The main goals are to determine the assumptions in factor analysis, create a method for identifying the factors, decide whether a factor is necessary, and investigate the interaction of the variables on the factor. One of the things the data set cannot have is the outliers. The data should have an adequate sample size and not have perfect multi-collinearity. Homoscedasticity is not required between variables, and it would be best to have the linearity of variables and interval data [50]. The descriptive statistics are the correlation matrix, Bartlett's test, sphericity, explained total variance, scree plot, and rotated component matrix. Factor analysis is often called data reduction.

Bartlett's test of sphericity tells that there is no normality; in other words, if it's significant, then the null hypothesis is rejected, which is that there's no departure from normality there [51]. A scree plot depicts how much these variations explain. It is noticed that variance one explained a bunch, variance two did a little more, and variance three explained a little more. And it gives us an eigenvalue, which correlates to the variance demonstrated. In factor analysis, an eigenvalue has to be one or more before its significant, and it has to be greater than or equal to 1.

The analysis produces a rotated component matrix, and factor loadings define the interaction of the variables among each factor.

Factor analysis examines the interrelationships between variables and provides a more meaningful summary presentation. There are two rotation methods, vertical and oblique, but the vertical rotation method is usually used. In addition, there are some prerequisites for performing factor analysis: the data set should be normally distributed and linear, data should be measurable, there should be no outliers in the data set, there should be no varying variance, there should be no multi-collinearity between the independent variables, a sample measurement should be sufficient. The sample size is tested through Kaiser-Meyer-Olkin (KMO) (1974). If the value obtained from the KMO test is more significant than 0.5, the sample size is assumed to be sufficient for factor analysis.

## 4 Results

Among the 28 independent variables included in the survey, certain groups in companies agreed on certain expressions, which means that if a participant indicated that they agree with an argument, they have probably decided, in other words. As a result, it is possible to compile statements that act simultaneously under more than one category, as specific characteristics of these claims in the survey may coincide in businesses. In addition, the survey looked at inconsistencies in responses and changes in business structure.

Employee competitiveness is critical if the characteristic is low (lower in quantity). Teamwork is also lacking, even though society does not support unorthodox conduct. Superiors do not always consult workers and are not always given instructions or follow formal processes. To be a good employee, you must do a good job. Colleagues lack confidence in one another, and they lack informal problem-solving skills. As a result, workers seldom share information. Idris and Kolawole [52], Cheung et al. [53], and Carrillo and Chinowsky [54] noticed the importance of a trusting environment that encourages employees to improve their skills. The equipment's long-term durability is not taken into account. The flow of information is complex, and decision-making systems work without the presence of people. They do not keep up with the markets via advisers; thus, networking and excellent relationships are essential.

If low (poor in quantity), performing a decent job isn't enough under this framework, and outstanding actions aren't valued. Employees do not have a high level of trust or competition. Workers are only loyal to the companies; they can't quickly leave. The workers have a say in the company's decisions. Positive connections with superiors do not ensure employment, and employees are unwilling to take on demanding positions to show their value. Equipment that will endure for a long time is wanted.

Femininity: Females are less noticeable when low (more in number). The respondents do not have the same qualifications, jobs, or salaries as men, and staff assignments are unclear. Formal standards for worker interactions and job management are expected if informality is low (higher in quantity). In a hierarchical culture, traditional rules and regulations, according to Cameron and Quinn [55], keep the organization together. Much work isn't enough to keep the system running correctly. While the organization is not strictly hierarchical, more clearly defined responsibilities exist. The companies often employ people in various areas, including administrative, technical, management, and site workers. Respondents' management positions were divided into three categories: top manager, manager of other managers, and manager of non-managers.

Respondents were required to provide the companies' represented work specialties to understand structural factors better. The respondents specified four work specialties: construction, construction management, engineering services, and project management. Employees in engineering services had the least competition; 82 percent of businesses worked in construction, while 20.4 percent worked in project management. The more workers a company had, the more gender-inclusive it was. The larger the company's workforce, the less casual and competitive the environment becomes.

Respondents were asked to describe their company's available and current market. Most organizations (84.2 percent) did not operate abroad, but some (15.8 %) did work abroad with international customers. The analysis was based on the number of accomplished projects by the firms in the last decade. A link was detected between the number of projects completed and the degree of femininity and informality. Female-friendly enterprises had completed 21–50 projects, and the businesses with 11–50 were the most formal. Many initiatives indicate a female-friendly mindset, but the atmosphere became male-dominated as the number exceeded 50.

Respondents were asked to provide associations to which their companies belonged to understand their businesses' specific conditions better. The most prominent trade organization was the Istanbul Chamber of Commerce, with 89.3 percent of the enterprises as members. The contracting businesses' primary activity may provide information about the companies' structure. The sector included two categories of contractors, with home building accounting for 49.5 percent of the total. The importance of general construction (39 percent) was likewise significant. The companies primarily engaged in general construction. The companies mainly involved in road and bridge construction welcomed women most. A limited number of businesses engaged in non-building construction were most concerned with certainty, as it was crucial to maintain control over the work. Competitiveness was the leading factor for firms classified as housebuilding types.

Businesses primarily serviced customers in the three spheres. 66.7 percent of the companies catered to private-sector customers. Approximately a quarter of construction firms (25.5 percent) work on private and governmental projects. The project type of the company corresponds to its business model. Businesses that only served the private sector were more competitive. According to the findings, housing was the most common business (87.9 %). Housing was the industry in which firms have shown the most competition. The inspection of work and staff and legal regulations mainly affected infrastructure enterprises.

### 4.1 Stages of factor analysis

First, the correlation between the observed variable values is calculated, and then the original variables become the correlation matrix (See Table 3). Many different factor derivation methods exist, but principal component analysis is generally used. The third step is converting the unrotated factor into a rotated one using the one-rotation method.

**Table 3:**
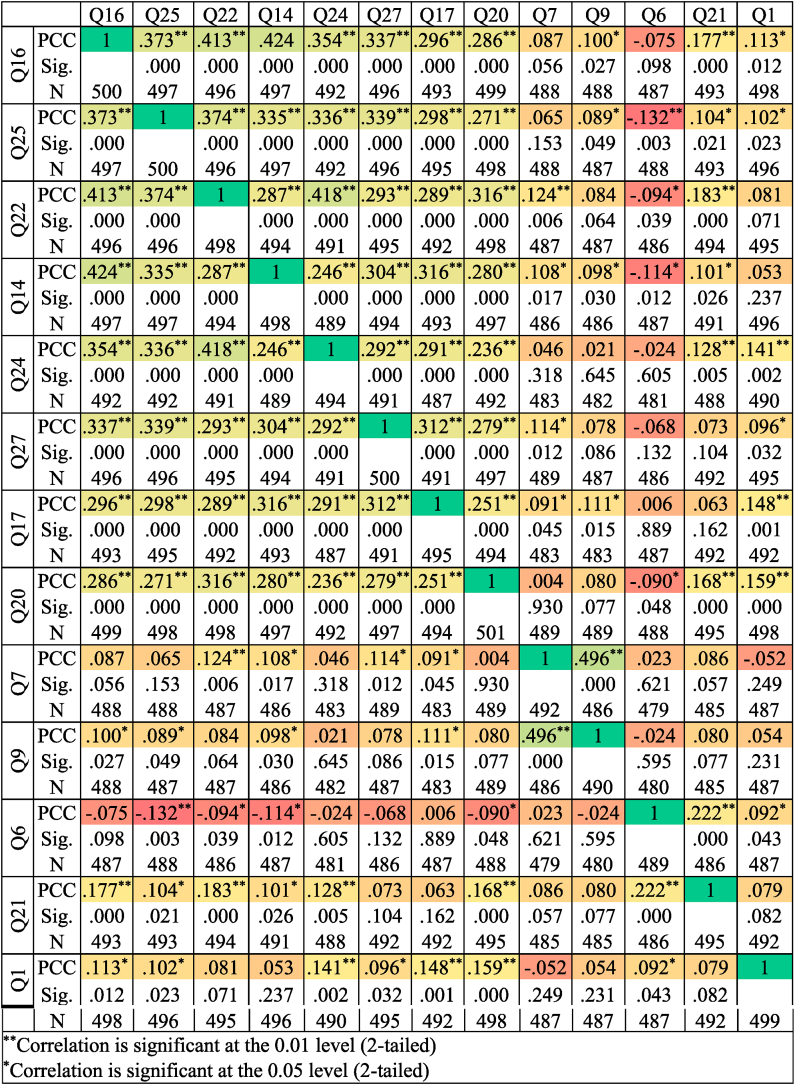
Pearson correlation analysis findings for the thirteen questions (Q): Pearson Correlation Coefficients (PCC) obtained are in the range of [-1,1].

Before starting the factor analysis, the dependent or independent variables are checked. When the correlation matrix table is examined, it is seen that the variables are dependent on each other. KMO value is 0.807 > 0.5 (See Table 4). By looking at this result, we can start the factor analysis.

**Table 4:** The KMO and Bartlett's tests include all the given information. A KMO score of more than 0.5 and Bartlett's test significance level of less than 0.05 indicate that the data are highly correlated.

Kaiser-Meyer-Olkin (KMO) and Bartlett's Test		
KMO Measure of Sampling Adequacy.		0.807
Bartlett's Test of Sphericity	Approx. Chi-Square	1002.739
	Df	78
	Sig.	0.000

**Fig. 2:**
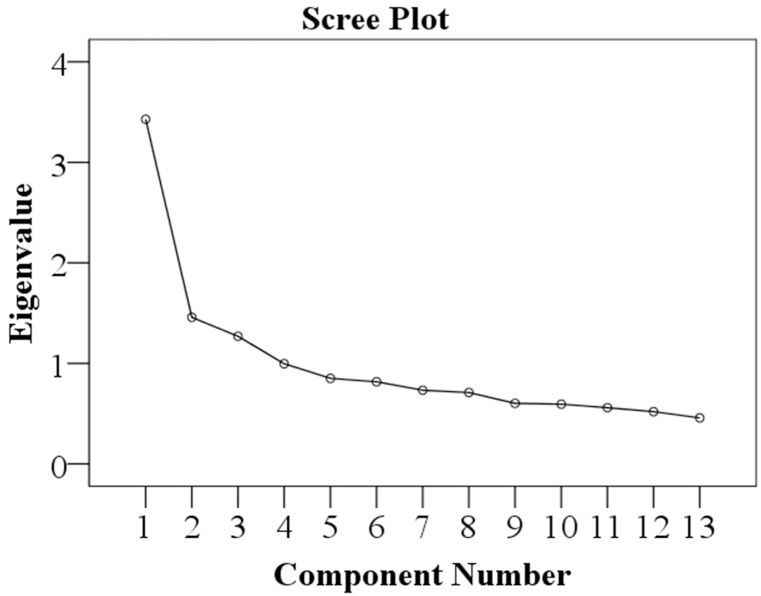
The scree plot determines the number of components included in exploratory factor analysis.

In this case, the sample size is sufficient. So, factor analysis can be done. Many methods are used in factor analysis to specify the optimal number of factors. It is adequate to define 2/3 of the variance with the variable included in the analysis; the variance should be 0.30. Factors with eigenvalues greater than one are considered. The Scree-Plot factor with the highest acceleration rapidly decreases, yielding the optimal factor number. The Scree plot provides an idea when determining the number of factors in factor analysis. There are points on the graph where the curve continues to flatten by counting how many factors there are (See Fig. 2).

When a variable has a cross-loading effect on factor analysis, researchers may disregard the troublesome variables and generate new factor solutions without them. Hence, 28 variables were reduced to 13 questions to avoid cross-loading.

Along with Fig. 2, if Table 5 of total variance through the principal component analysis is interpreted, how many absolute values are above 1.000 in the scale is apparent. Three factors explain approximately 47.37 % of the total variance in this case; hence, three components were extracted, as seen in Table 5.

**Table 5:** The amount of variation described by a factor is assessed in eigenvalues, and whether a component is retained is determined by the sum of the squared loadings.

Total Variance Explained									
Component	Initial Eigenvalues			Extraction Sums of Squared Loadings			Rotation Sums of Squared Loadings		
	Total	% of variance	Cumulative %	Total	% of variance	Cumulative %	Total	% of variance	Cumulative %
**1**	3.429	26.380	26.380	3.429	26.380	26.380	3.328	25.598	25.598
**2**	1.458	11.219	37.599	1.458	11.219	37.599	1.519	11.682	37.280
**3**	1.270	9.771	47.371	1.270	9.771	47.371	1.312	10.090	47.371
4	0.997	7.667	55.038						
5	0.851	6.547	61.585						
6	0.817	6.285	67.870						
7	0.733	5.639	73.510						
8	0.710	5.464	78.974						
9	0.603	4.636	83.610						
10	0.594	4.570	88.180						
11	0.559	4.304	92.484						
12	0.520	3.997	96.482						
13	0.457	3.518	100.000						

Extraction Method: Principal Component Analysis.

The correlation coefficient between the original variables and the component is called component loadings in Principal Component Analysis (PCA). In Table 6, it can be seen that all the variables are gathered under three different factors. It is seen in the table that the factor loads in the first factor are in the range of approximately 0.552–0.708, the factor loads in the second factor are in the range of about 0.795–0.830, and the factor loads in factor three are in the range of approximately 0.579–0.787.

**Table 6:** The correlations between items and components, also known as factor loadings, are shown in the component matrix.

Component Matrix^a^			
	Component		
	1	2	3
Q16. It is often vital to develop documented formal processes to ensure the completion of the project.	0.708		
Q25. Colleague's suggestions for improvement are greatly appreciated.	0.675		
Q22. Employees in our organization discuss difficulties and keep their co-workers up-to-date on significant developments.	0.671		
Q14. Our workers are concerned about their supervisors' reactions to their everyday jobs, particularly in unusual circumstances.	0.617		
Q24. Tax advisors or management consultants significantly impact our company's growth.	0.607		
Q27. Some of my co-workers have risen through the ranks due to their experience.	0.593		
Q17. Only essential and long-lasting equipment is purchased in our organization. Therefore, employees are asked to use caution while using them.	0.581		
Q20. We get the most remarkable outcomes at our organization by working together.	0.552		
Q7. Equally competent women do the same tasks as men.		0.830	
Q9. Working alongside a male or female colleague, whether superiors or subordinates, makes no difference.		0.795	
Q6. It is uncommon to change the company's future or how a particular function is performed, leaving co-workers astonished.			0.787
Q21. Some of our staff are unique individuals with unusual attitudes and working styles.			0.642
Q1. Our organization has written, unwritten, and informal regulations and every co-worker is aware of them and expects others to follow their lead.			0.579

Extraction Method: Principal Component Analysis.

a three components extracted.

**Fig. 3:**
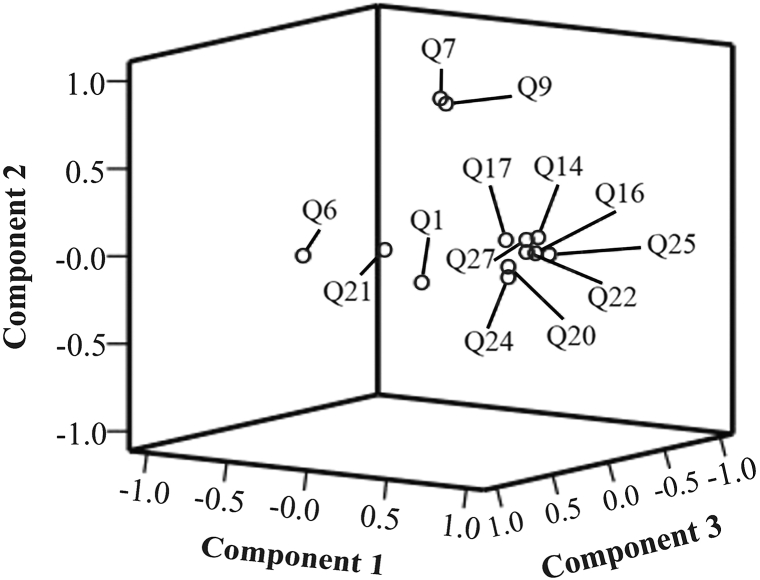
The components in the space-rotated graph offer a more precise presentation in which the position of data concerning the link between data conveys information on data similarities.

The positive and negative component loadings show the variables' positive and negative correlation with the P.C.s. If the aim is looking for a linear connection, the negative numbers indicate the direction of the correlation between the component and the variable since the correlation may be positive or negative. Furthermore, the cutoff criteria should be determined whether the value is negative or absolute while shortlisting the components. Finally, the factor's dependability is determined by the connection between the individual rotating factor loading and the final sample size magnitude (See Fig. 3).

The most important strategy used in the research to assess the cultural behavior patterns that significantly impact the construction industry's operation is to survey the workers to get information about their cultural habits.

Following a literature review, eight dimensions and twenty-eight variables, referred to as indicators, were identified. We obtained sufficient surveys from interviews with construction industry employees to obtain a statistically significant result. It may be advantageous that such surveys may be done in a shorter time without compromising statistical reliability. Therefore, a statistical analytic approach known as factor analysis should determine which variable or indicator will be eliminated. Exploratory factor analysis was utilized in this investigation.

As a result of the analysis, thirteen out of twenty-eight variables were adequate for conducting the research and obtaining statistically significant findings. According to the study results, thirteen variables are divided into three components in eight dimensions (See Table 7). Describing three components as independent dimensions, each covering eight dimensions, is also feasible. As a result, thirteen variables were eventually identified under three dimensions: multiple motivational, one–bipolar, and many bipolar dimensions, instead of the previously described eight dimensions (See Table 8).

**Table 7:** After the factor analysis, dimensions and engaged questions were grouped into a simplified questionnaire.

Dimension 1. Universalism/Particularism [39,43]
Factor 3 – Q1. Our organization has written, unwritten, and informal regulations and every co-worker is aware of them and expects others to follow their lead.
**Dimension 2**. **Power Distance** [25,46]
**Factor 3 – Q6.** It is uncommon to change the company's future or how a particular function is performed, leaving co-workers astonished.
**Dimension 3**. **Masculinity/Femininity** [25,46]
**Factor 2 – Q7.** Equally competent women do the same tasks as men.
**Factor 2 – Q9.** Working alongside a male or female colleague, whether superiors or subordinates, makes no difference.
**Dimension 4**. **Uncertainty Avoidance** [25,46]
**Factor 1 – Q14.** Our workers are concerned about their supervisors' reactions to their everyday jobs, particularly in unusual circumstances.
**Factor 1 – Q16.** It is often vital to develop documented formal processes to ensure the completion of the project.
**Dimension 5**. **Long Term Orientation** [25,46]
**Factor 1 – Q17.** Only essential and long-lasting equipment is purchased in our organization. Employees are asked to use caution while using them.
**Dimension 6**. **Individualism/Communitarianism** [39,43]
**Factor 1 – Q20.** We get the most remarkable outcomes at our organization by working together.
**Factor 3 – Q21.** Some of our staff are unique individuals with unusual attitudes and working styles.
**Factor 1 – Q22.** Employees in our organization discuss difficulties and keep their co-workers up-to-date on significant developments.
**Dimension 7**. **Inner-Directed/Outer-Directed** [39,43]
**Factor 1 – Q24.** Tax advisors or management consultants significantly impact our company's growth.
**Factor 1 – Q25.** Colleague's suggestions for improvement are greatly appreciated.
**Dimension 8**. **Achieved Status/Ascribed Status** [39,43]
**Factor 1 – Q27.** Some of my co-workers have risen through the ranks due to their experience.

**Table 8:** Core categories emerged during component analysis, and a consequent questionnaire was created referencing Freeman and Bordia's [47] dimensions.

Core dimension 1. Multiple motivational dimensions [47]
**Factor 1 – Q14.** Our workers are concerned about their supervisors' reactions to their everyday jobs, particularly in unusual circumstances.
**Factor 1 – Q16.** It is often vital to develop documented formal processes to ensure the completion of the project.
**Factor 1 – Q17.** Only essential and long-lasting equipment is purchased in our organization. Employees are asked to use caution while using them.
**Factor 1 – Q20.** We get the most remarkable outcomes at our organization by working together.
**Factor 1 – Q22.** Employees in our organization discuss difficulties and keep their co-workers up-to-date on significant developments.
**Factor 1 – Q24.** Tax advisors or management consultants significantly impact our company's growth.
**Factor 1 – Q25.** Colleague's suggestions for improvement are greatly appreciated.
**Factor 1 – Q27.** Some of my co-workers have risen through the ranks due to their experience.
**Core dimension 2**. **One–bipolar dimensions** [47]
**Factor 2 – Q7.** Equally competent women do the same tasks as men.
**Factor 2 – Q9.** Working alongside a male or female colleague, whether superiors or subordinates, makes no difference.
**Core dimension 3**. **Many bipolar dimensions** [47]
**Factor 3 – Q1.** Our organization has written, unwritten, and informal regulations and every co-worker is aware of them and expects others to follow their lead.
**Factor 3 – Q6.** It is uncommon to change the company's future or how a particular function is performed, leaving co-workers astonished.
**Factor 3 – Q21.** Some of our staff are unique individuals with unusual attitudes and working styles.

## 5 Discussion

In recent years, the study of the link between organizational culture and performance has grown tremendously [44,53,56]. Organizational performance improvement has been found to result from translating values and beliefs into policies and practices. Unsurprisingly, an organization's culture directly impacts its execution ability [42,57]. A significant and persuasive effect on employee behavior may be achieved by highlighting specific ideals and developing appropriate standards for anticipated behavior [58].

The outcome of this study suggests that particularism has a considerable presence in the sector. Employees emphasize that their organization has a clear hierarchy at work, where decisions are made at a high level, and managers rarely ask for people's thoughts at lower levels. The positions that female workers hold in the industry, the rights they have to engage in talks about their company's future or the achievement of their employment, and their level of remuneration all show a company's masculinity or femininity. The poll has 11.9 percent female participation, indicating that the dominant gender in the field is male.

A unique fear of uncertainty implies that colleagues at all levels of management, for example, rarely make choices on their own. The polar opposite would be an attitude in which decision-making is highly valued and making mistakes is more easily tolerated. Uncertainty is avoided as much as possible in the industry while supervising staff is not required due to the confidence created among co-workers. Several steps are made to prevent uncertainty from occurring. First, formal procedures are precisely stated so there is no ambiguity about how people can exercise initiative. Second, it implies that mistakes are not tolerated and that employees are always aware that superiors make decisions. Third, either workers' or enterprises' market behaviors are likely to be within the long-term perspective. The sector's planning horizon is broad, and keeping what they have for the long haul will be helpful in the future. As a result, the structure is appropriated using this method. Finally, individualism implies that individuals have distinct personality traits, but communitarianism implies that the overall functioning of the group is essential compared to the attitude and behavior of individual employees. Individualistic actions would take up less space because everyone has a say in making consensus-based decisions. Inner-directed means that a corporation develops ideas and plans with its staff, whereas Outer-directed means implies that it is quickly impacted by developments gathered outside of the company.

External conditions and current trends are vital in tailoring the organization to the market, but internal solutions provided by personnel are also valued. Finally, businesses would take advantage of any opening to enter the market. Achieved Status denotes that individuals in your organization are promoted based on the quality of their work. Ascribed status implies that transactions, relationships, a good name, or specific privileges are essential. One's qualifications are more significant than having particular networks to gain access.

Traditional procurement is the primary method used for non-housing buildings, although Traditional Design and Build methods are equally used for housing. Project management is more commonly employed in civil engineering projects. Traditional norms and regulations keep an organization together in a hierarchical society. The larger a company's workforce, the more gender-inclusive it was. There was a correlation between the number of finished projects and the degree of femininity and informality. The Istanbul Chamber of Commerce was the most influential trade group. Workers in the construction business are concerned about their bosses' reactions to their daily tasks, especially in exceptional circumstances. To compete in the construction industry, particularly at the organizational level, building project organizations and contracting firms must understand human behavior and culture, according to the findings, primary contractors' control, and direct sector dynamics. Turkish businesses frequently hire workers in various positions, including administrative, technical, managerial, and site-worker positions. The management positions of respondents were classified into three categories: top manager, manager of other managers, and manager of non-managers. The larger a company's workforce, the more gender-inclusive it was. Female-friendly businesses completed 21–50 initiatives, while companies with 11–50 were the most formal. Engineering managers must comprehend the relationship between organizational culture and the successful completion of building projects. Managers must be aware that corporate models vary from one another. The larger a company's workforce, the more gender-inclusive it was. There was a correlation between the number of finished projects and the degree of femininity and informality. Tax advisors and management consultants significantly impact the company's growth, and employees are advised to use their prudence. High financial statistics can assure that all conditions for completing construction projects are met, but they cannot guarantee that all organizations will meet established targets.

This study aims to examine the significance of components derived from 28 statements. A factor analysis, a statistical analytical method, was used to determine which variable, the indicator, would be deleted. It was concluded that thirteen out of twenty-eight would be sufficient to perform the research and generate statistically meaningful results.

The researchers employed principal component analysis to examine cultural behavior patterns that substantially impact the construction industry's operation. When creating a new scale, exploratory factor analysis is used. In general, factor analysis informs us about designing the survey's sub-parameters. When creating a new scale, exploratory factor analysis is used. Generally, factor analysis tells us how to prepare the survey's sub-parameters. Factors are not directly observed; variables are followed, and factors develop from variables. Colleagues lack trust in one another and have informal problem-solving abilities.

Employees have a low level of trust and competition. The obtained correlation coefficients are in the range [−1,1] - with a factor of 1.065. As a result, factor analysis is possible. When a variable has a cross-loading effect on factor analysis, researchers may ignore the problematic variables and produce new factor solutions in their absence. Three factors account for approximately 47.37 percent of the total variance. The variance a factor describes is evaluated in eigenvalues, and the sum of the squared loading determines whether a component is maintained. In general, the dependability of the factor is governed by the relationship between the individual rotating factor loading and the absolute sample size magnitude. The investigation was carried out to examine cultural behavior patterns that substantially impact the construction industry's operation. In this study, exploratory factor analysis was used. Dimensions and engaging questions were gathered into a more straightforward questionnaire using factor analysis. According to the study's findings, thirteen variables are separated into three components within eight dimensions. Hence, dimensions and engaging questions were gathered into a more straightforward questionnaire using factor analysis.

### 5.1 Theoretical contribution

Organizational learning has become a vital talent that all businesses should have. Organizational learning is a dynamic knowledge production, acquisition, and integration process that aims to improve organizational performance by developing resources and capacities.

To foster a learning environment, firms should establish a culture that supports employee communication, encourages experimentation and risk-taking, and inspires workers to examine core ideas and work habits. In addition, increasingly competitive environments need a greater focus on individual and organizational performance, which may be strongly impacted.

The research makes a significant theoretical addition by highlighting the organizational behavior that is affected by the culture of the stakeholders in the Turkish construction sector. This research used correlation analysis and discovered a substantial statistical link between the questionnaire items used to evaluate corporate culture's impact on performance.

The research also makes a substantial theoretical addition by demonstrating that a broader viewpoint on disclosing the critical cultural aspects has been surveyed using a quantitative technique, Factor Analysis, and Principal Component Analysis with the fewest possible questions.

The theoretical contribution of our study is that our findings assist in explaining the underlying mechanisms by which organizational culture affects construction project success. Current research is one of the most comprehensive studies examining Turkish organizational culture at the contracting business and organizational levels. Results show that the construction sector may be developed following organizational culture.

### 5.2 Engineering managers' implications

Organizational culture establishes the blueprint for how a company achieves its goals and is thus critical to its success. Practicing engineering managers must recognize that the organizational culture aids in materializing technical talents to produce results. The internal operational standards, construction methods, conventions, and written-down regulations that guide every corporation's action are all part of the culture in this case.

Organizational culture is derived from societal practices. Any conflict between the organization's operations and societal norms, which could work against the organization, must be prevented. Managers should also be aware that corporate models differ from one to the next and determine what each firm values most. Construction managers need to understand the connection between the organizational culture and the effective execution of construction projects since the organizational culture shapes members' perceptions, directs their actions, and connects internal processes with external demands to establish a compatible relationship with all elements impacting construction [53].

Referring to the professional engineering managers, all contractual construction agreements, procurement methods, and the usage of building materials are all founded on a company's culture, which will help safeguard the company against fraud and eliminate the need for management to guess. Assume that engineering management is unaware of the corporate culture. In such a situation, disputes and opposition from numerous sources, such as the community, workers, and other trustworthy departments directly impacting the building, such as procurement and finance, can limit overall success.

## 6 Conclusion

This study provides a framework to deepen our understanding of contracting organizations' cultural variability. This study could broaden its scope to include other stakeholders in the Turkish construction industry, such as architectural design and engineering firms, by investigating the project participants' unique cultural attributes, orientations, behaviors, and motives. As a result, decision-makers or engineering managers must play a variety of responsibilities to ensure practical project completion and competitiveness. A leader or project manager may use this document to understand cultural strengths and weaknesses before implementing the management components of construction projects.

An empirical framework based on the Turkish construction sector's cultural orientation is presented in this research. The empirical study leads to an understanding of contracting firms' culture and organizational behavior in two ways: For starters, it created an evaluation model for organizational culture based on predetermined dimensions. Second, the study accepted vital cultural characteristics in contracting firms.

In the Turkish construction industry, the most common position of the firms' responders was mainly primary contractors. Findings suggest that primary contractors are in charge of controlling and coordinating sector dynamics. The private sector is responsible for most construction projects, demonstrating that construction enterprises are firmly embedded in the sector's dynamics. Clients of the most recently completed projects, in other words, are from the private sector. As a result, contractor companies' large number of private projects leads to an economic advantage that gives them a favorable position.

The most significant impediment to responding to survey questions is the time it takes to complete the questionnaire. Therefore, designing and implementing a questionnaire that will yield the most reliable results with the fewest questions possible while still being easy to administer is vital. Since it could be favorable that such surveys can be administered much shorter without sacrificing statistical reliability, a factor analysis, a statistical analysis method, was applied to decide which variable, the indicator, will be eliminated. Exploratory factor analysis was involved in this study. As a result of the analysis, it was determined that thirteen out of twenty-eight variables would be sufficient to conduct the research and to obtain statistically significant results.

Furthermore, it has been determined that thirteen variables constitute three factors in the context of eight dimensions, and it is also possible to express three factors as separate dimensions covering eight dimensions. Therefore, thirteen variables were finally defined under three dimensions: multiple motivational, one–bipolar, and many bipolar, instead of eight. Consequently, it has become possible to successfully quantify the cultural behaviors of employees in the construction sector associated with sectoral success using a more compact survey due to this research.

## Data availability and ethics statement

Some or all data, models, or codes that support the findings of this study are available from the corresponding author upon reasonable request. This research was approved by the Akdeniz University Scientific Research and Publication Ethics Board with 309356 on March 04, 2021.

## CRediT authorship contribution statement

**Muhammed Ernur Akıner:** Writing – review & editing, Writing – original draft, Visualization, Validation, Supervision, Software, Resources, Project administration, Methodology, Investigation, Formal analysis, Data curation, Conceptualization. **İlknur Akıner:** Writing – review & editing, Writing – original draft, Visualization, Validation, Supervision, Software, Resources, Project administration, Methodology, Investigation, Formal analysis, Data curation, Conceptualization. **İbrahim Yitmen:** Writing – review & editing, Writing – original draft, Visualization, Validation, Supervision, Software, Resources, Project administration, Methodology, Investigation, Formal analysis, Data curation, Conceptualization.

## Declaration of competing interest

The authors declare that they have no known competing financial interests or personal relationships that could have appeared to influence the work reported in this paper.
